# The use of DNA repair genes as prognostic indicators of gastric cancer

**DOI:** 10.7150/jca.31062

**Published:** 2019-08-27

**Authors:** Chang Jinjia, Wang Xiaoyu, Sun Hui, Li Wenhua, Zhang Zhe, Zhu Xiaodong, Xu Midie

**Affiliations:** 1 Department of Medical Oncology, Fudan University Shanghai Cancer Center, Shanghai, 200032, China; 2 Experiment Center for Science and Technology, Shanghai University of Traditional Chinese Medicine, Shanghai 201203, China; 3 Department of Pathology, Fudan University Shanghai Cancer Center, Shanghai, 200032, China; 4 Department of Pathology & biobank, Fudan University Shanghai Cancer Center, Shanghai, 200032, China

## Abstract

DNA repair genes can be used as prognostic biomarkers in many types of cancer. We aimed to identify prognostic DNA repair genes in patients with gastric cancer (GC) by systematically bioinformatic approaches using web-based database. Global gene expression profiles from altogether 1,325 GC patients' samples from six independent datasets were included in the study. Clustering analysis was performed to screen potentially abnormal DNA repair genes related to the prognosis of GC, followed by unsupervised clustering analysis to identify molecular subtypes of GC. Characteristics and prognosis differences were analyzed among these molecular subtypes, and modular key genes in molecular subtypes were identified based on changes in expression correlation. Multivariate Cox proportional hazard analysis was used to find the independent prognostic gene. Kaplan-Meier method and log-rank test was used to estimate correlations of key DNA repair genes with GC patients'overall survival. There were 57 key genes significantly associated to GC patients' prognosis, and patients were stratified into three molecular clusters based on their expression profiles, in which patients in Cluster 3 showed the best survival (P < 0.05). After a three-phase training, test and validation process, the expression profile of 13 independent key DNA repair genes were identified can classify the prognostic risk of patients. Compared with patients with low-risk score, patients with high risk score in the training set had shorter overall survival (P < 0.0001). Furthermore, we verified equivalent findings by these key DNA repair genes in the test set (P < 0.0001) and the independent validation set (P = 0.0024). Our results suggest a great potential for the use of DNA repair gene profiling as a powerful marker in prognostication and inform treatment decisions for GC patients.

## Introduction

Gastric Cancer (GC), including the gastro-esophageal junction (GEJ) adenocarcinoma, is one of the most common cancers worldwide 1. In China, the disease burden of GC is extremely high. Previous study reported that an estimated 4,292,000 new cancer cases and 2,814,000 cancer deaths occurred in China in 2015 2. Surgery, chemotherapy, and molecular targeted therapies are the mainstay of treatment for GC patients. Most patients, however, are diagnosed at an advanced stage and, therefore, miss operation chance 3. Therefore, detection of potential prognostic biomarker is crucial for GC patients prognosis and treatment.

DNA repair is a collection of processes by which cells identify and correct damages to the DNA molecules that encode its genome. The reaction may restore the DNA structure to its original function, whereas sometimes it cannot completely eliminate DNA damage and only enable cells to tolerate and survive with DNA damages. When normal repair processes fail without activation of cellular apoptosis, irreparable DNA damage may occur 4. This can eventually lead to cellular senescence and even malignancies in human entities. DNA damage can be caused by both extracellular (e.g. radiation and virus) and intracellular (e.g. reactive oxygen) environmental factors and normal metabolic processes. Helicobacter pylori is a well-established risk factor of GC and may cause injuries to genomic integrity through an inefficient DNA repair 5.

DNA damage repair, proceed by several mechanisms, including base excision repair, mismatch excision repair, nucleotide excision repair and homologous recombination, is crucial to maintaining the integrity of the genome and the exercise of normal function. Variations in DNA repair capacity resulting from somatic mutations could therefore correlate with GC progression 6. DNA damage caused by internal and external factors can be repaired by DNA repair genes, which involving in activation or inhibition of specific DNA repair related pathways. Multiple genes that were initially shown to influence life span have turned out to be involved in DNA damage repair and protection 7. Large-scale genome sequencing of GC indicated that somatic mutations in genes involved in homologous recombination DNA repair are common features 8, 9. A thorough understanding of the DNA repair genes expression profile in tumor tissues would be shed new light on cancer prognostication and improvement of therapeutic response.

In this present study, we selected 1,325 GC samples from the Gene Expression Omnibus (GEO) and The Cancer Genome Atlas (TCGA) GC database, aimed to clarify the relationship between the expression patterns of GC associated DNA repair genes and GC prognoses. Different molecular subtypes were built for further identification of the correlation between gene expression and GC prognosis. With repeated unsupervised clustering analysis, multivariate Cox proportional hazard analysis and Robust likelihood-based modeling, 13 key prognostic DNA repair genes were finally identified for GC. Our study provided a group of biomarker that identifies the subset of GC deficient in DNA gene repair, which is critical for the rational design of clinical trials using DNA mismatch repair targeting agents.

## Materials and Methods

### Data download and preprocessing

The Microarray gene expression profiles of gastric cancer, GSE33335, GSE27342, GSE63089, GSE62254 and GSE26253, were downloaded from Gene Expression Omnibus database (GEO http://www.ncbi.nlm.nih.gov/geo/). The general information of each dataset was shown in **Supplemetary Table 1**. All patients in all six datasets were staged by the 7th of the AJCC gastric cancer staging system 10. Formalin fixed paraffin embedded (FFPE) tissues were used in GSE26253 and GSE62254, and frozen tissue specimens were used in GSE33335, GSE27342, GSE63089 and the TCGA GC datasets. On the basis of maximum possible inclusion strategy, a total of 727 DNA repair genes were obtained from KEGG and reported in previous articles (http://www.cgal.icnet.uk/DNA_Repair_Genes.html, up to June 2017) 11 **(Supplementary Table 2)**. Actually, there were many outdated genesymbol, that is, one specific gene matched to different names in the 727 gene lists, so finally we obtained 215 genes with expression level in the learning set (GSE62254).

The expression profile data were matched to all genes and all no-load probes were removed. The median value is selected if multiple probes matched to a single gene, and further quantile standardization was used to standardize each dataset, finally the expression profile of DNA repair genes were extracted.

### Analysis expression profiles of DNA repair genes

The expression profiles of DNA repair genes in cancer and adjacent normal tissue samples were analyzed in GSE33335, GSE27342 and GSE63089 datasets, GSEA (hallmarks sets_BP) was implemented by using R survival software, to observe the GO biological processes that DNA repair genes involved.

### Screening of DNA repair genes which related to prognosis

Cancer samples at different stages may show different DNA gene expression patterns, which is closely related to patients' prognosis. We analyzed DNA repair gene expression profile of each sample in the GSE62254 dataset and classified samples using hierarchical clustering, then analyzed the differences on patients' prognosis between different categories. Given that patients' prognosis altered with different gene expression levels, we selected DNA repair genes whose expression dysregulation variance is greater than 0.1 for further analysis. Potentially altered genes, which were screened from patient samples, were separately analyzed using univariate COX proportional hazards model. Genes with P-values less than 0.05 were selected.

### GC molecular subtype construction and prognostic genes analysis

GC molecular subtypes were built by unsupervised clustering of prognostic DNA repair genes. The Kaplan-Meier curve with log-rank analysis was used for prognosis analysis of each molecular subtypes and patients with different risk. Clinical characteristics were identified and compared through observing individual subtypes. The expression correlation among these genes was analyzed by Pearson correlation coefficient.

### Independent prognostic genes and formula exploring

We did a multivariate Cox regression analysis using a backward stepwise approach to test if the gene was an independent prognostic factor of overall survival, and derived a formula to calculate the risk score for every patient from the expression values of independent prognostic DNA repair genes, weighted by regression coefficient. Risk score=expgene1*βgene1+expgene2*βgene2+...expgene10*βgene10 (exp: expression level, β: the regression coefficient derived from the multivariate Cox regression model). We used receiver operating characteristics (ROC) curves to analyze the sensitivity, specificity and Youden index for the prediction of survival by the DNA repair gene signature 12.

### Verifying of key prognostic genes through external data

The influence of DNA repair genes on prognosis was verified in GSE26253 and TCGA GC dataset, respectively. The RNA sequence data of gastric cancer in The Cancer Genome Atlas (TCGA) was downloaded from (http://cancergenome.nih.gov/). A total of 433 patient samples and their clinical follow-up information were available.

### Statistical analysis

All statistical analyses were done with R survival package two-tailed tests, and significance was defined as p values of less than 0.05 13.

## Results

### Sample data download and pre-processing

**Figure 1:**
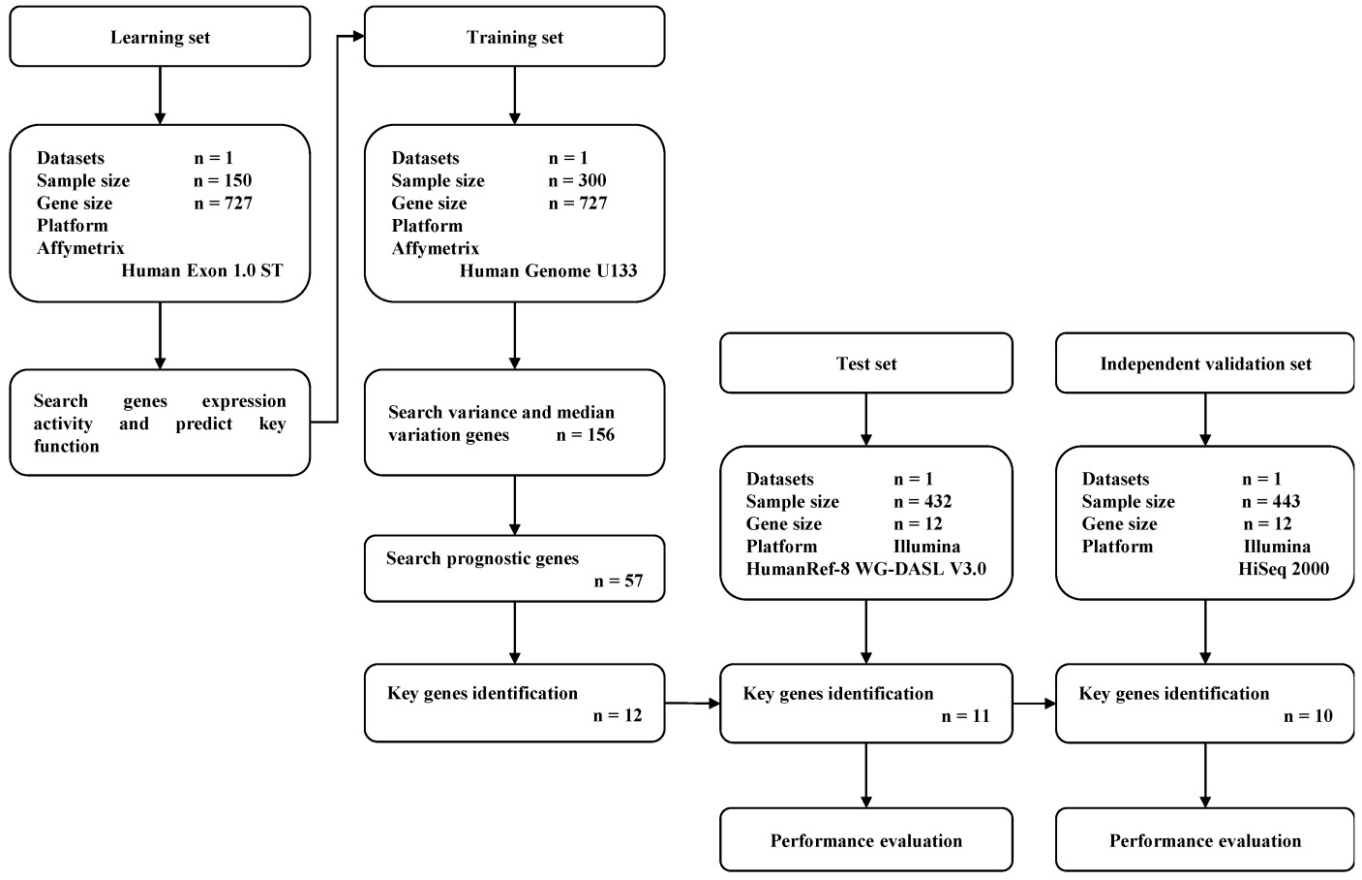
Flow diagram of gene expression signature identification and performance evaluation.

A total of 1,325 GC samples which included 23,521 gene expression values were obtained from 5 GEO GC datasets (GSE33335, GSE27342, GSE63089, GSE62254 and GSE26253) and the TCGA GC dataset. A total of 727 expressed DNA repair genes were selected from these GC samples. The flow diagram of key prognostic gene identification and performance evaluation was shown in **Figure 1**.

### Expression pattern of DNA repair genes in GC and adjacent normal tissues

**Figure 2:**
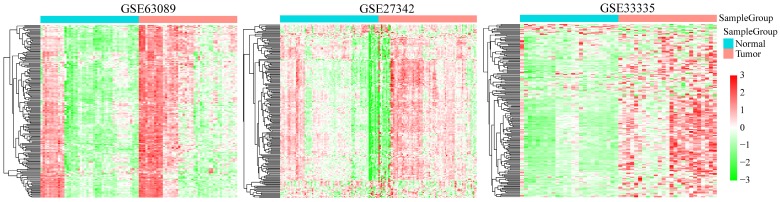
Clustering analysis of 727 DNA repair genes in indicated GEO GC database. The horizontal axis represents sample, using Euclidean distance to calculate distance; the vertical axis stands for genes, using Pearson correlation coefficient to calculate distance.

We analyzed the expression profiles of DNA repair genes in three groups of cancer and adjacent normal tissues (GSE33335, GSE27342 and GSE63089). As shown in **Figure 2**, the average expression level of DNA repair gene in GC samples was higher than that in adjacent tissues, suggesting DNA repair was more active in cancer tissues than normal GC tissues.

In order to observe the biological processes associated with the DNA repair genes in GC, we used GSEA database (GO gene sets) to analyze DNA repair gene expression enrichment in these three GEO GC datasets. As shown in **Supplementary Figure 1** and** Supplementary Table 3**, they were significantly enriched in several biological processes including cell cycle (GO cell cycle G1/S phase transition and GO cell cycle phase transition), metabolism (GO DNA metabolic process) and related molecular function (GO positive regulation of molecular function). It is suggested that cell cycle abnormalities in GC tissues were associated with DNA repair genes.

### Relationship between different expression patterns of DNA repair genes and GC prognosis

We analyzed the expression profiles of DNA repair genes in GSE62254 dataset (in which we extracted 215 DNA repair genes) by hierarchical clustering and categorized the expression profiles of these DNA repair genes into three groups (group IV was excludes because it only has one sample), in which Group III showed the lowest overall DNA repair activity while Group II showed the highest overall DNA repair activity (**Supplementary Figure 2A**). Next, we analyzed the overall survival of patients in these three groups, which showed that the patient in Group III had a significantly worst outcome than those patients in Group I (P < 0.0001,** Supplementary Figure 2B**) and Group II (P = 0.0021, **Supplementary Figure 2C**). This indicates that DNA repair genes could significantly distinguish GC patients' prognostic outcomes.

**Figure 3:**
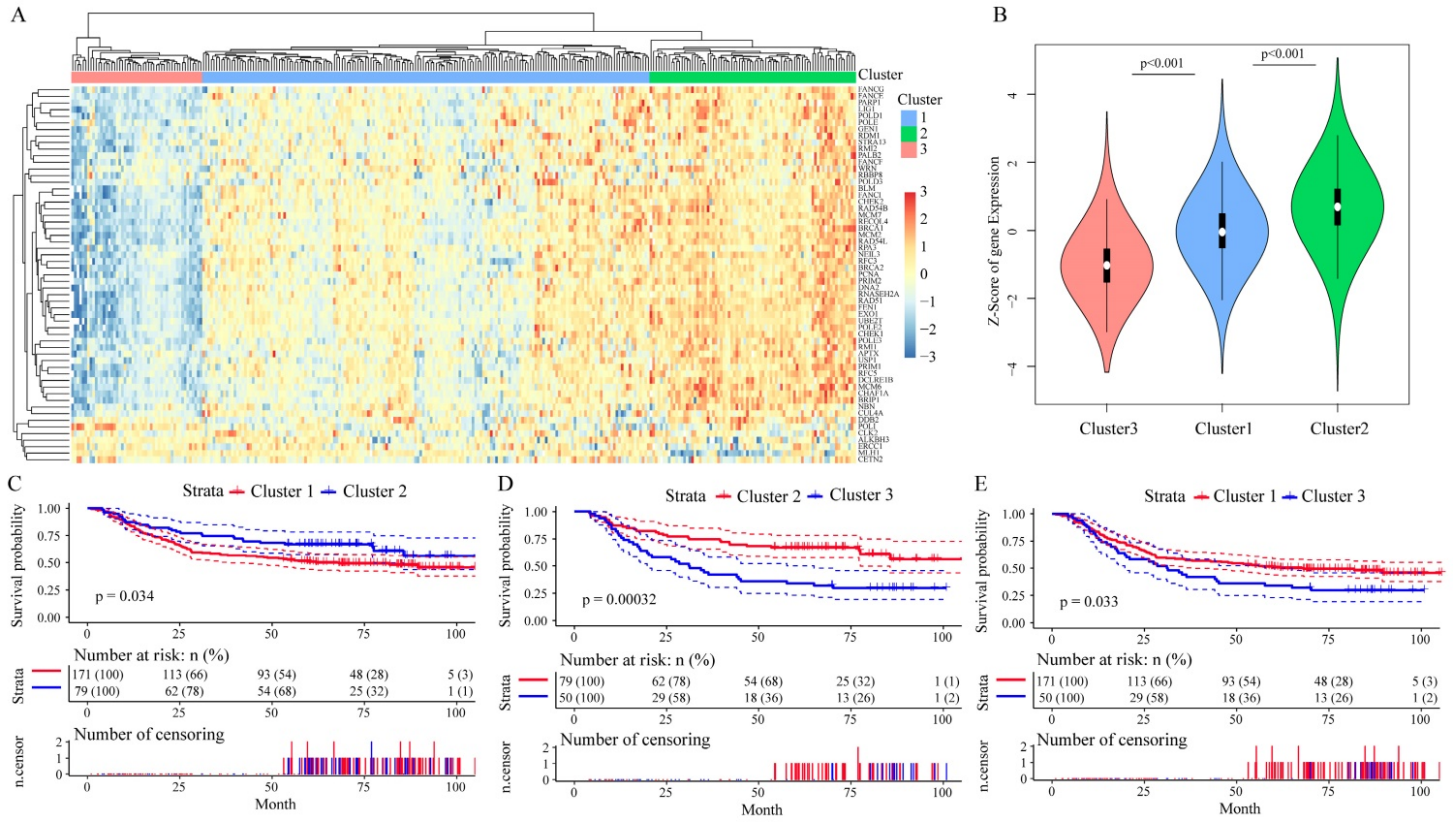
The prognostic effect of 57 DNA repair genes on GC patients' prognosis in the GSE62254 dataset. A. Clustering analysis of DNA repair gene expression profile which significantly influence prognosis, horizontal axis divides the sample into 2 groups; B. Expression of DNA repair genes in indicated clusters; C-E. Kaplan-Meier method and log-rank test of the prognosis difference between patients in indicated clusters.

Among these 215 genes in the GSE62254 dataset, we enrolled 156 genes whose variance > 0.1 in all samples for Cox univariate survival analysis and got 57 genes with significant influence on GC patients' prognosis (all P < 0.05, **Table 1**), in which the Hazard ratio (HR) of 52 genes were less than 1. We then analyzed the correlation among the expression level of these 57 prognostic genes by Pearson correlation coefficient, the hierarchical cluster of correlation between each gene showed that 50 of the 57 DNA repair genes were positively correlated with each other, while 7 genes had negative correlations (**Supplementary Figure 3**). By performing unsupervised clustering of these 57 DNA repair genes, we divided GC patients into three clusters (**Figure 3A**), and DNA repair gene expression significantly differed in these three clusters (**Figure 3B**). Moreover, patients Cluster 2 had a better outcome than patients in Cluster 1 and Cluster 3 (P < 0.05, **Figure 3C-E**). The analysis of clinicalpathological characteristics distribution of the three groups is shown in **Supplementary Figure 4**. Based on this analysis, the age distribution of groups III was significantly lower than group I (P = 0.0002) and group II (P < 0.0001). This suggests that the better prognosis of groups II should be age-related.

### Development of a prognostic risk score formula using DNA repair genes in GC

We further identified 13 independent prognostic genes by Cox multivariate survival analysis from these 57 DNA repair genes, and derived a formula to calculate the risk score for each patient from the expression values of the 13 DNA repair genes, weighted by regression coefficient. The specific formula for the GSE62254 dataset was as follows: Risk score = (0.41 * expMCM2 + 0.2 * expMLH1 + 0.25 * expCLK2 - 0.34 * expFANCG + 0.38 * expEXO1 - 0.33 * expPARP1 + 0.29 * expCETN2 - 0.25 * expNEIL3 + 0.24 * expALKBH3 + 0.24 * expPOLI - 0.18 * expPOLD3 + 0.22 * expERCC1 - 0.29 * expFANCF). The forest map of these 13 genes is shown in **Supplementary Figure 5**, in which five genes (FANCG, PARP1, NEIL3, POLD3 and FANCF) showed HR < 1, and eight genes (MCM2, MLH1, EXO1, CLK2, CETN2, ALKBH3, POLI and ERCC1) showed risk HR > 1.

**Figure 4:**
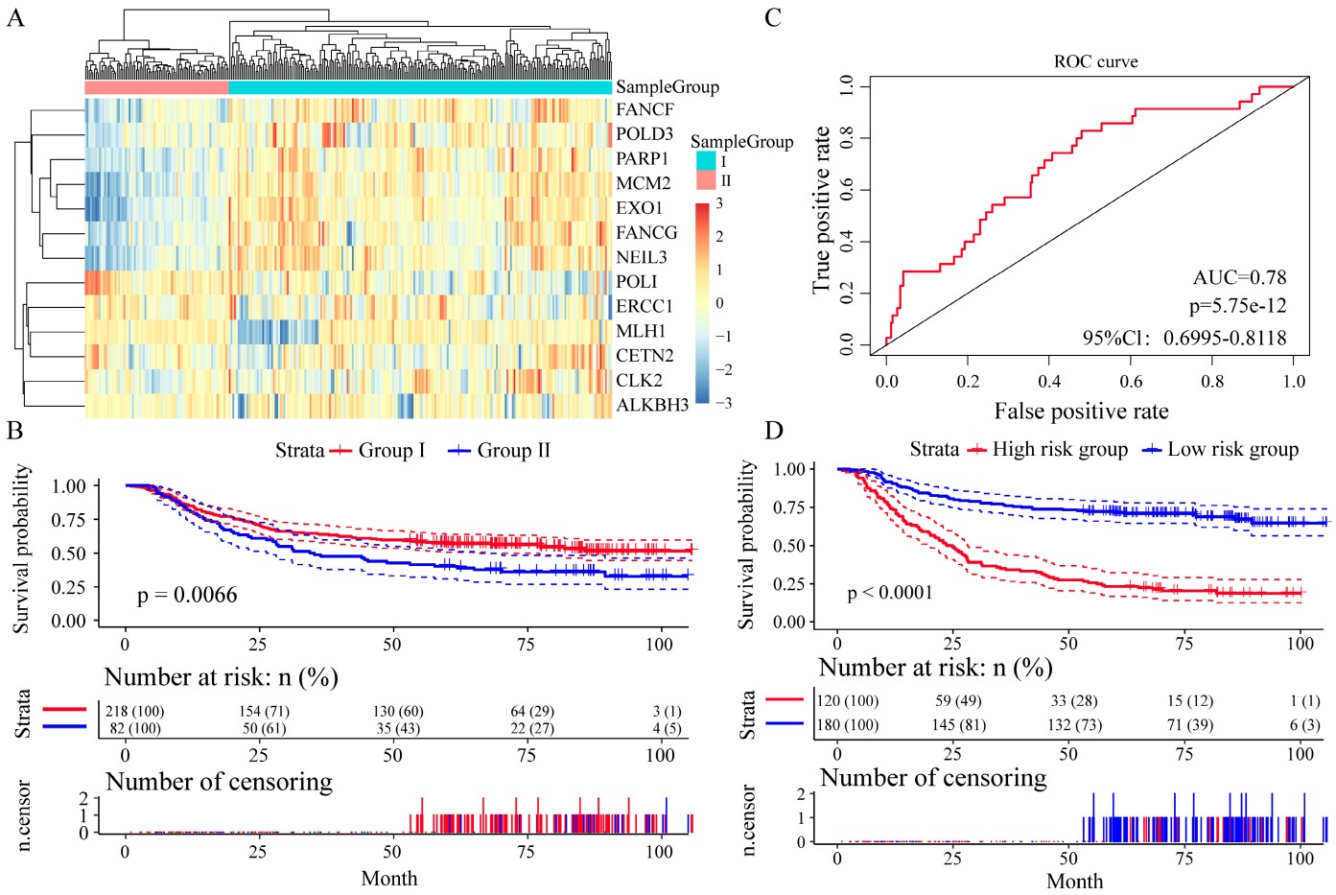
Relationship between different expression patterns of 13 key DNA repair genes and GC prognosis in the training set. A. Clustering analysis of 13 key DNA repair gene expression profile, horizontal axis divides the sample into 2 groups; B. Kaplan-Meier method and log-rank test of the prognosis difference between patients in indicated clusters; C. Receiver operating characteristics (ROC) curves for overall survival in the training set. P values show the area under the ROC (AUROC) of 13 DNA repair genes gene signature; D. Kaplan-Meier method and log-rank test of the prognosis difference between patients in indicated group.

We divided the patients into two groups (group I and group II) by the expression spectrum clustering analysis, and found that the expression level of seven genes (FANCF, POLD3, PARP1, MCM2, EXO1, FANCG and NEIL3) in Group I was higher than that in Group II (**Figure 4A**). Compared with patients in group I, patients in group II had significantly shorter overall survival (P = 0.0066, **Figure 4B**). In Receiver operating characteristic (ROC) curve to analyze sensitivity and specificity of survival prediction, the area under the AUC line was 0.780 (P = 5.7528e-12, **Figure 4C**). With this risk score formula, patients in the learning set were divided into high-risk or low-risk groups with the Youden index (0.2446) as the cutoff. Compared with patients in the low-risk group, patients in the high-risk group had shorter overall survival (P < 0.0001, **Figure 4D**).

### Independent datasets test and validation

**Figure 5:**
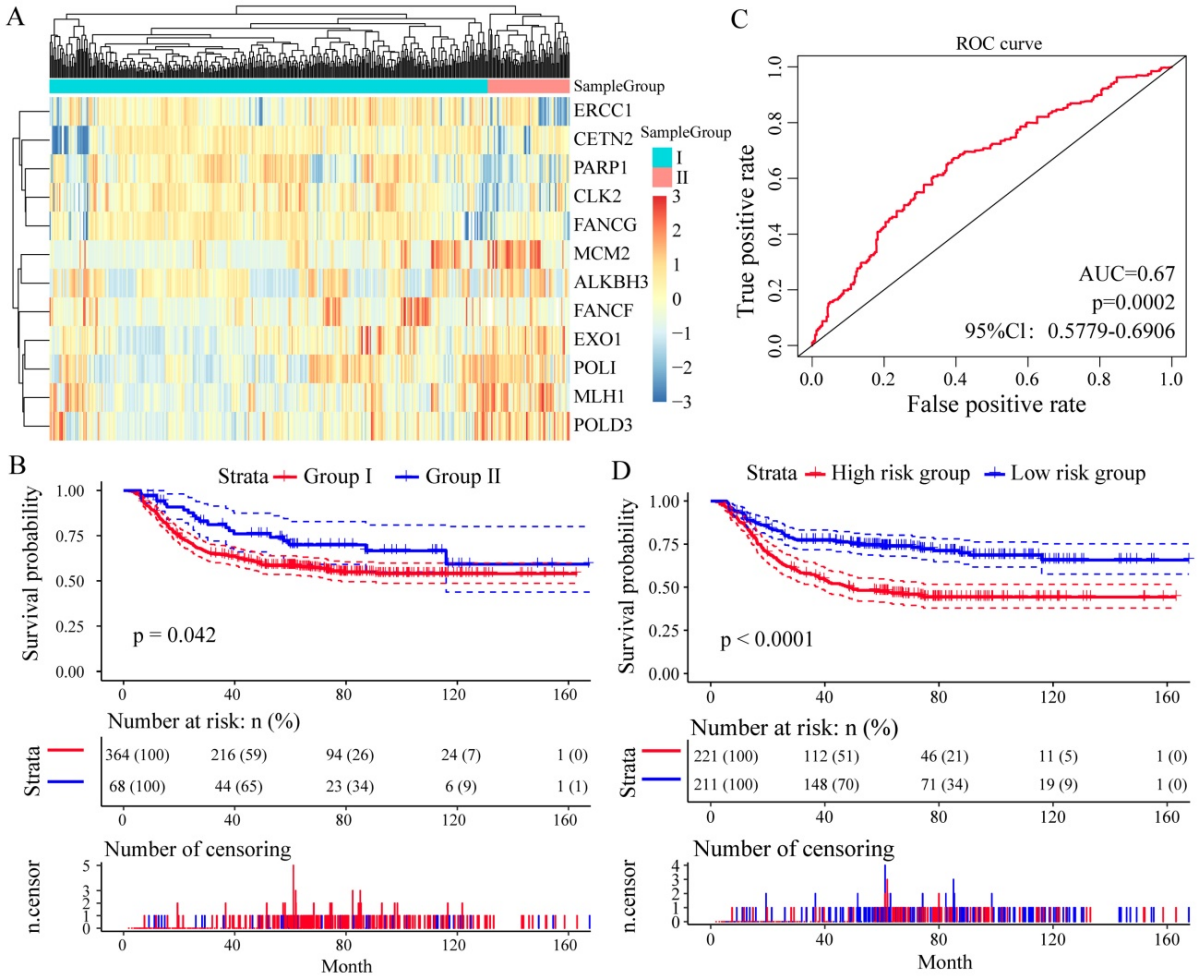
Relationship between different expression patterns of 13 key DNA repair genes and GC prognosis in the test set. A. Clustering analysis of 13 key DNA repair gene expression profile, horizontal axis divides the sample into 2 groups; B. Kaplan-Meier method and log-rank test of the prognosis difference between patients in indicated clusters; C. Receiver operating characteristics (ROC) curves for overall survival in the training set. P values show the area under the ROC (AUROC) of 13 DNA repair genes gene signature; D. Kaplan-Meier method and log-rank test of the prognosis difference between patients in indicated group.

To assess whether these 13 DNA repair genes had the same or similar prognostic value in different populations, we extracted the expression profiles of 13 DNA repair genes in the test datasets GSE26253 (n = 432), 12 genes (except of NEIL3) were found. With the same algorithm used in the learning set, we developed a new formula for the expression data from the 432 GC tissues from the test set as follow: Risk score = (-0.1003 * expMCM2 - 0.0247 * expMLH1 - 0.1412 * expCLK2 - 0.1752 * expFANCG - 0.4061 * expEXO1 +0.1372 * expPARP1 + 0.1547 * expCETN2 + 0.0116 * expALKBH3 - 0.0509 * expPOLI - 0.0159 * expPOLD3 + 0.1722 * expERCC1 - 0.1339 * expFANCF). By the expression spectrum clustering analysis, we also divided the patients into two groups (Group I and Group II, **Figure 5A**), and compared with patients in Group I, patients in Group II had significant shorter overall survival (P = 0.042, **Figure 5B**). In ROC analysis, the area under the AUC line was 0.671 (P = 0.0002, **Figure 5C**). With this risk score formula, patients in the test set were divided into high-risk or low-risk groups with the Youden index (3.9377) as the cutoff. As expected, patients in the test set with high risk-scores had shorter overall survival than those with low risk-scores (P < 0.0001, **Figure 5D**).

**Figure 6:**
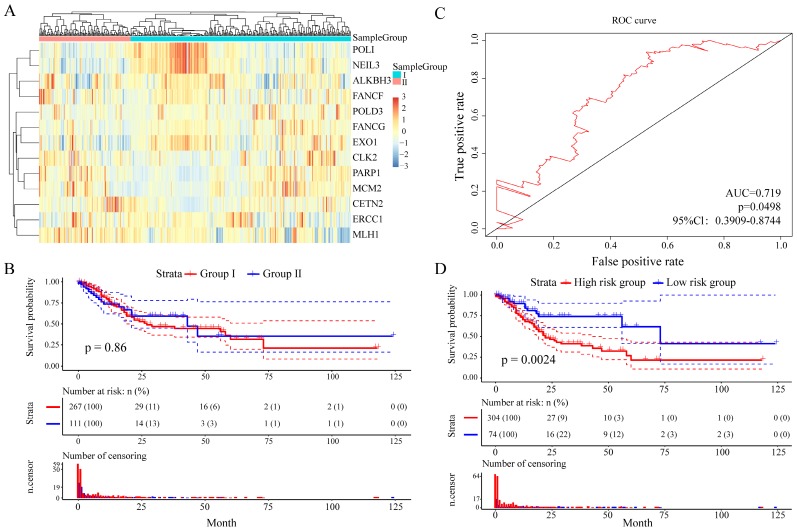
Relationship between different expression patterns of 13 key DNA repair genes and GC prognosis in the independent validation set. A. Clustering analysis of 13 key DNA repair gene expression profile, horizontal axis divides the sample into 2 groups; B. Kaplan-Meier method and log-rank test of the prognosis difference between patients in indicated clusters; C. Receiver operating characteristics (ROC) curves for overall survival in the training set. P values show the area under the ROC (AUROC) of 13 DNA repair genes gene signature; D. Kaplan-Meier method and log-rank test of the prognosis difference between patients in indicated group.

And then we further assess the prognostic value of these 13 DNA repair genes in an independent validation set (TCGA GC dataset, n = 443). With the same algorithm used in the learning set, we developed a new formula for the expression data from the 443 GC tissues from the independent validation set: Risk score = (-0.9094 * expERCC1 - 0.1896 * expCETN2 - 0.2124 * expPARP1 -1.70426 * expCLK2 - 0.1481 * expFANCG - 1.3774 * expMCM2 + 0.9026 * expALKBH3 - 2.1348 * expFANCF + 5.4205 * expEXO1 - 1.4920 * expPOLI + 1.5893 * expMLH1 - 2.8104 * expPOLD3 - 6.6587 * expNEIL3). By the expression spectrum clustering analysis, we also divided the patients into two groups (**Figure 6A**), whereas there was no significant difference on overall survival between group I and group II (P = 0.86, **Figure 6B**). In ROC analysis, the area under the AUC line was 0.719 (P = 0.0498, **Figure 6C**). With this risk score formula, patients in the learning set were divided into high-risk or low-risk groups with the Youden index (0.1516) as the cutoff. As expected, patients in the independent validation set with high risk-scores had shorter overall survival than those with low risk-scores (P = 0.0024, **Figure 6D**). The clinicalpathological characteristics distribution between the two groups divided by the 13 DNA repair genes showed significantly differences **(Supplementary Figure 6)**.

## Discussion

Cumulative studies have reported DNA damage repair deficiencies in multiple solid tumor types, including GC 14, 15. DNA repair genes are differentially expressed in normal tissues and cancer tissues, the abnormal expression of DNA repair genes in human cancers is significantly associated with the patients' prognosis 9, 16, 17. The existence of tumor heterogeneity and complexity specificity, to some extent, greatly limits the prognostication value (specificity, reproducibility and generalisability) of individual gene 18, 19. Thus, employing a gene expression panel instead of an individual gene as a biomarker provides a rational option to circumvent the limitation in genes utilisation in predicting GC outcomes. However, the globe expression profile of DNA repair genes and their clinical application prospect has yet to be elucidated in GC. In the current study, we identified an expression profile of 13 independent key DNA repair genes which could classify the prognostic risk of patients.

With the development of microarray and next-generation sequencing technology, increasing data resources regarding DNA repair genes, such as TCGA and GEO databases, are available for comparing and analysing in the human malignancies 20, 21. In the current study, bio-information of gene spectrum in 150 GC samples obtained from three GEO GC datasets, was analyzed to determine the expression activity of DNA repair genes in GC. As a result, the expression activity of 727 DNA repair genes we examined were significantly higher in GC samples compared with normal gastric mucosa, which indicated that DNA damage repair is a common event in GC tumorigenesis and progression. Therefore, their expression levels may be an indicator of the intrinsic characteristics of GC. DNA repair genes could potentially be sensitive and specific prognostic predictors. Indeed, we confirmed that the less active of DNA repair genes expression in cancer samples, the worse the prognosis. All this suggested that patients with higher DNA repair gene expression abundance tend to have a better outcome and most of the DNA repair genes were prognostic protective factors.

Unsupervised clustering analysis further verified that the prediction by using the molecular signature of these prognostic effective DNA repair genes is matched to the clinical outcomes for these samples. The expression profile of DNA repair genes in cluster 2 were significantly higher than the others two clusters. And the overall survival of cluster 2 was significantly higher than of the others two clusters, indicated that most of the 57 genes may be the promoters of DNA damage repair and tumor suppressor in GC. Interestingly, the differentially expressed DNA repair genes described here, including POLD3, MLH1, MCM2 and ERCC1, are similar to those listed in previous reports 22-25. These aforementioned studies, together with our results, firmly support the notion that DNA repair gene expression profile may generate a unique molecular signature for prognostication of GC.

The most striking results came from the analysis of 13 DNA repair genes (MCM2, MLH1, CLK2, FANCG, EXO1, PARP1, CETN2, NEIL3, ALKBH3, POLI, POLD3, ERCC1 and FANCF) whose altered expression profile was significantly related to the survival of GC patients. DNA damage repair has been list as the main process involving in cell cycle, and specific genetic changes to a great extent abrogate the fidelity of DNA replication 26. Our GESA analysis results also proposed that the dysregulated DNA repair genes in GC are enriched in cell cycle related biological process. Thus, the reason that these 13 genes appear to have a prognostic impact on the survival could be due to the pivotal cell cycle regulating activities of these genes on tumor biology. POLD3 and MLH1, who were implicated in epigenetic alterations of DNA mismatch repair (MMR), are associated with tumorigenesis of GC by causing microsatellite instability 22, 23. The origin licensing factor minichromosome maintenance 2 (MCM2) expression variation could act as an independent prognostic indicator for patients with diffuse-type GC 17. Moveover, low expression of excision repair cross-complementation group 1 (ERCC1) was a significant independent favorable prognostic factor in patients with advanced GC who were receiving first-line chemotherapy regardless of the treatment regimen in JCOG9912 27. Although the further phase III study did not meet its endpoint, the results of GOLD study reported that patients with DNA repair-deficient tumors who exhibiting loss of expression of the ataxia-telangiectasia mutated (ATM) protein might benefit from PARP1 inhibitor treatment 28. MLH1 methylation was related to oxaliplatin resistance in GC patients, which providing the potential of MLH1 to be utilized as a chemo-sensitive marker 16. Our results agree with the observations that MCM2, MLH1 and ERCC1 may serve as an oncogene and that PARP1 may function as tumor suppressors genes during tumorigenesis. Nevertheless, additional studies to investigate how the altered expression of these 13 genes contribute to the tumorigenesis and/or progression of GC would improve our understanding of the molecular basis of this tumor, and might ultimately lead to novel therapeutic interventions, as well as prognostic tools for this disease.

We also enrolled the TCGA GC dataset for validation of the prognostication ability of these 13 key DNA repair genes, whereas patients in the molecular group didn't reach a constantly effective value. Given the patient's heterogeneity of the multi-center dataset is much bigger than that of single center datasets, we applicated Cox regression analysis and Youden index to increase the prediction value.

In conclusion, we describe the first, to our knowledge, use of DNA repair gene to assess their ability on GC classification that improves the current disease stratification. The biological relevance of these subtypes is illustrated by significant differences in prognosis. Our study provides possibilities for improving prognostic models and therapeutic strategies of GC patients. The limitation of the current study was lack of validation in a large prospective patient cohort. Moreover, we did not consider the different treatment pattern and their impact on patient survival in this study. Future prospective clinical trials are warranted to further consolidate the validity of the 13-gene signature.

## Supplementary Material

Supplementary figures and tables.

## Figures and Tables

**Table 1:** The 57 DNA repair genes with significant prognosis value in the GSE62254 dataset

Number	Gene symbol	P.Value	Low 95%CI	High 95%CI	HR
1	FEN1	0.0001	0.2349	0.1127	0.4896
2	POLE2	0.0001	0.2925	0.1557	0.5493
3	RAD51	0.0002	0.1658	0.0647	0.4249
4	RMI1	0.0003	0.1137	0.0350	0.3697
5	CETN2	0.0003	6.4920	2.3457	17.9673
6	NEIL3	0.0006	0.3399	0.1841	0.6275
7	PRIM2	0.0006	0.2237	0.0946	0.5289
8	POLD3	0.0007	0.1081	0.0301	0.3883
9	BRCA1	0.0007	0.1403	0.0452	0.4354
10	CHAF1A	0.0010	0.1436	0.0454	0.4540
11	RNASEH2A	0.0010	0.2618	0.1180	0.5808
12	DNA2	0.0011	0.2859	0.1346	0.6076
13	POLE3	0.0012	0.1654	0.0558	0.4907
14	CUL4A	0.0013	0.1184	0.0321	0.4362
15	CHEK1	0.0014	0.3356	0.1717	0.6560
16	BRCA2	0.0015	0.3049	0.1466	0.6342
17	FANCG	0.0019	0.2342	0.0938	0.5851
18	PCNA	0.0019	0.2521	0.1056	0.6018
19	BRIP1	0.0020	0.3026	0.1419	0.6455
20	FANCF	0.0020	0.1612	0.0507	0.5129
21	NBN	0.0020	0.2677	0.1160	0.6178
22	APTX	0.0021	0.1558	0.0478	0.5085
23	EXO1	0.0025	0.4001	0.2210	0.7245
24	RMI2	0.0030	0.3176	0.1491	0.6765
25	POLI	0.0031	4.3131	1.6367	11.3660
26	RDM1	0.0037	0.2473	0.0963	0.6355
27	UBE2T	0.0038	0.4194	0.2328	0.7556
28	MLH1	0.0038	2.9212	1.4120	6.0434
29	MCM6	0.0039	0.2250	0.0816	0.6204
30	ERCC1	0.0050	6.8881	1.7895	26.5132
31	FANCI	0.0052	0.3851	0.1971	0.7521
32	RAD54B	0.0057	0.3803	0.1916	0.7547
33	GEN1	0.0061	0.1539	0.0404	0.5858
34	POLD1	0.0064	0.2750	0.1088	0.6951
35	RAD54L	0.0070	0.2814	0.1120	0.7074
36	RECQL4	0.0075	0.1846	0.0535	0.6376
37	RBBP8	0.0097	0.2968	0.1183	0.7447
38	POLE	0.0122	0.1745	0.0445	0.6837
39	WRN	0.0124	0.2499	0.0843	0.7407
40	RFC3	0.0124	0.5452	0.3390	0.8770
41	LIG1	0.0128	0.2492	0.0834	0.7444
42	DCLRE1B	0.0139	0.2523	0.0842	0.7557
43	PARP1	0.0140	0.1868	0.0490	0.7123
44	CLK2	0.0146	5.7213	1.4102	23.2119
45	STRA13	0.0151	0.2459	0.0793	0.7627
46	RFC5	0.0152	0.2980	0.1121	0.7919
47	MCM2	0.0164	0.4399	0.2250	0.8602
48	PRIM1	0.0165	0.3864	0.1776	0.8406
49	PALB2	0.0176	0.1780	0.0428	0.7402
50	DDB2	0.0287	0.3717	0.1532	0.9021
51	MCM7	0.0310	0.4196	0.1906	0.9236
52	CHEK2	0.0324	0.3932	0.1672	0.9248
53	ALKBH3	0.0368	2.5888	1.0603	6.3211
54	RPA3	0.0405	0.3501	0.1283	0.9555
55	BLM	0.0453	0.4881	0.2418	0.9851
56	USP1	0.0476	0.3651	0.1347	0.9893
57	FANCE	0.0491	0.3104	0.0968	0.9953

## Abbreviations

GC: gastric cancer
GEO: Gene Expression Omnibus
TCGA: The Cancer Genome Atlas
ROC: receiver operating characteristics
HR: hazard ratio
FUSCC: Fudan University Shanghai Cancer Center
