# Supplementary material for: The use of DNA repair genes as prognostic indicators of gastric cancer
Source: J Cancer. 2019 Aug 27;10(20):4866–75. doi: 10.7150/jca.31062 (PMC6775511; doi:10.7150/jca.31062)
Supplement: Supplementary file 1 — Supplementary figures and tables. [file jcav10p4866s1.pdf]

Supplementary Figure 1

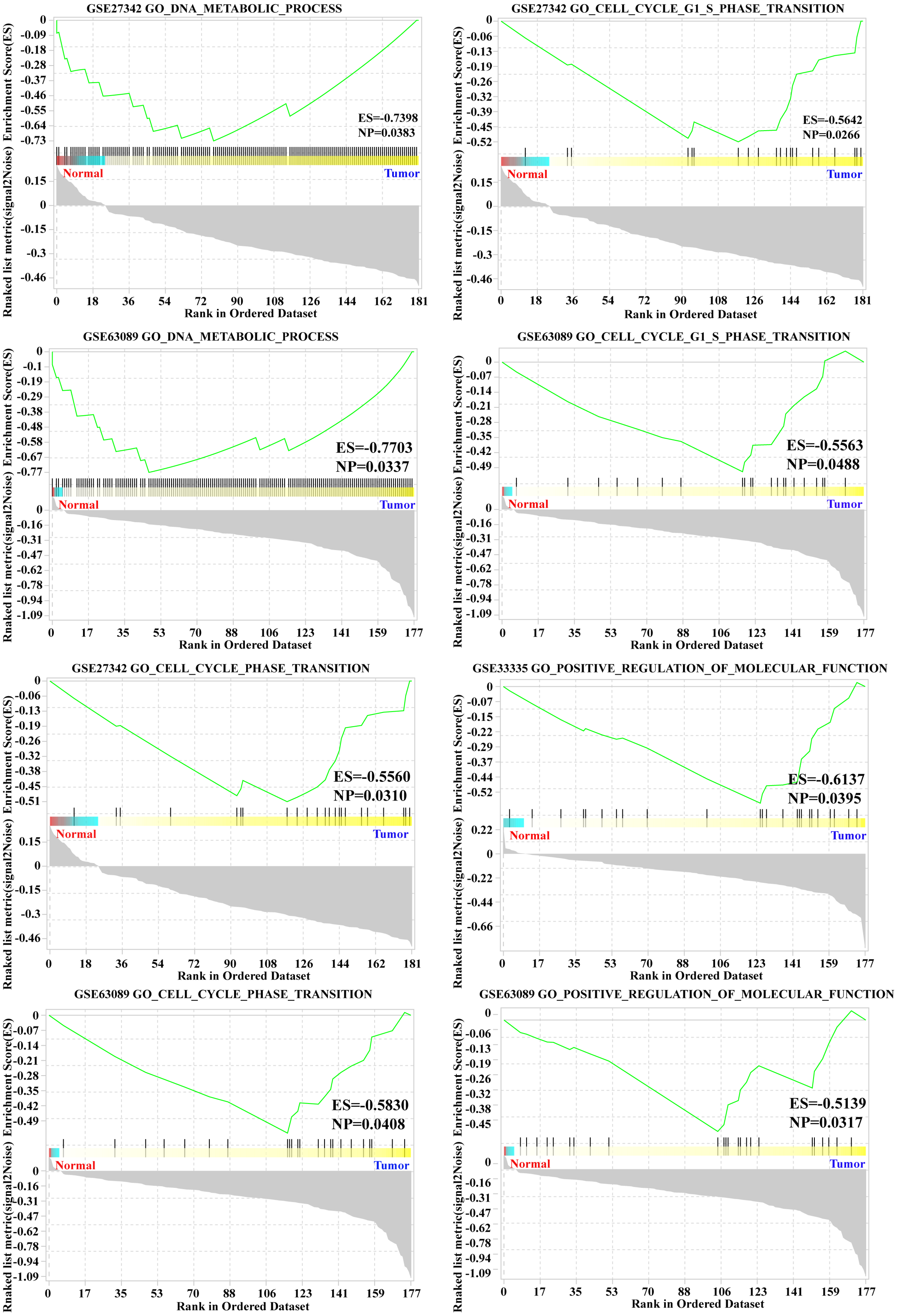

Supplementary Figure 2

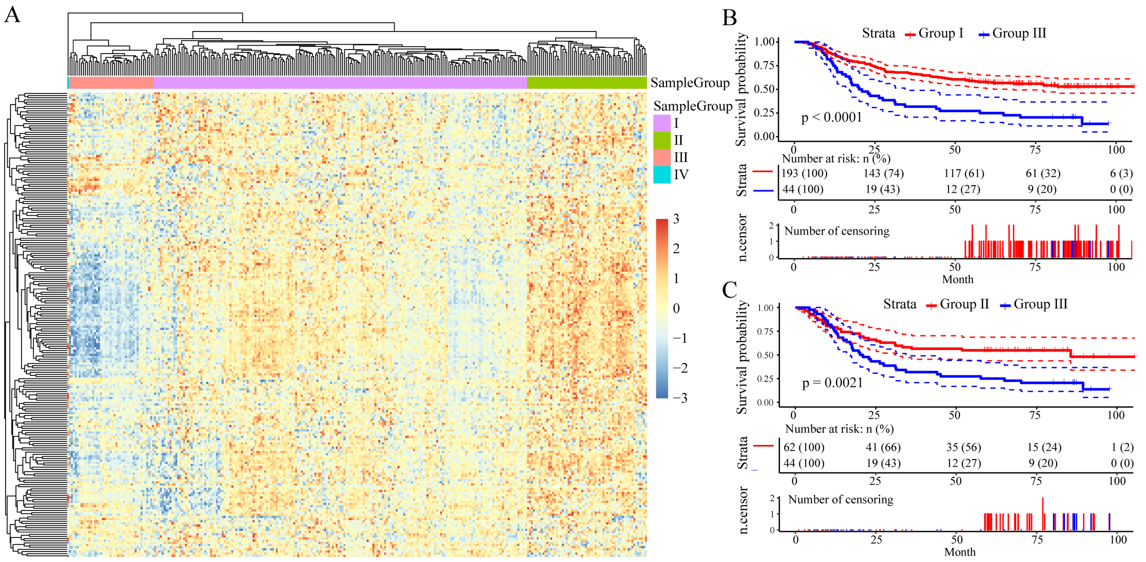

Supplementary Figure 3

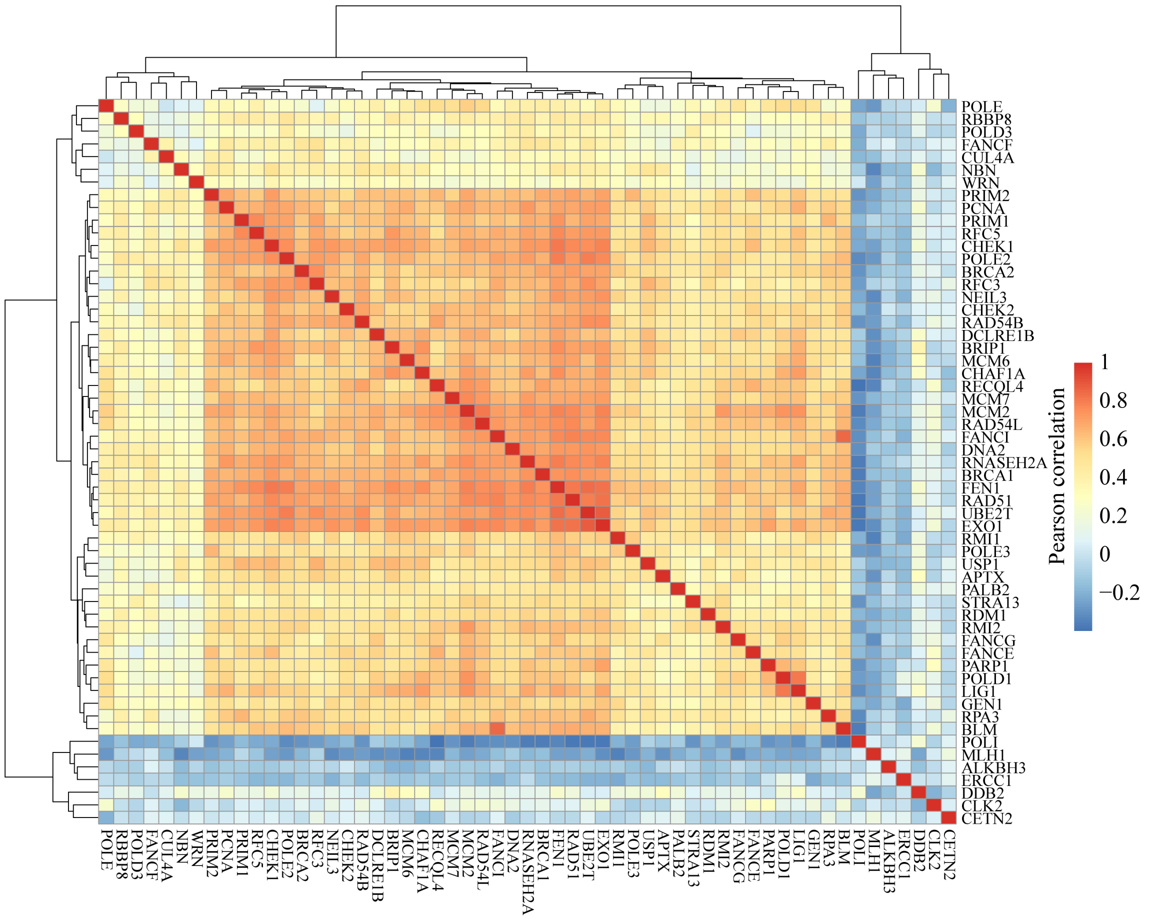

Supplementary Figure 4

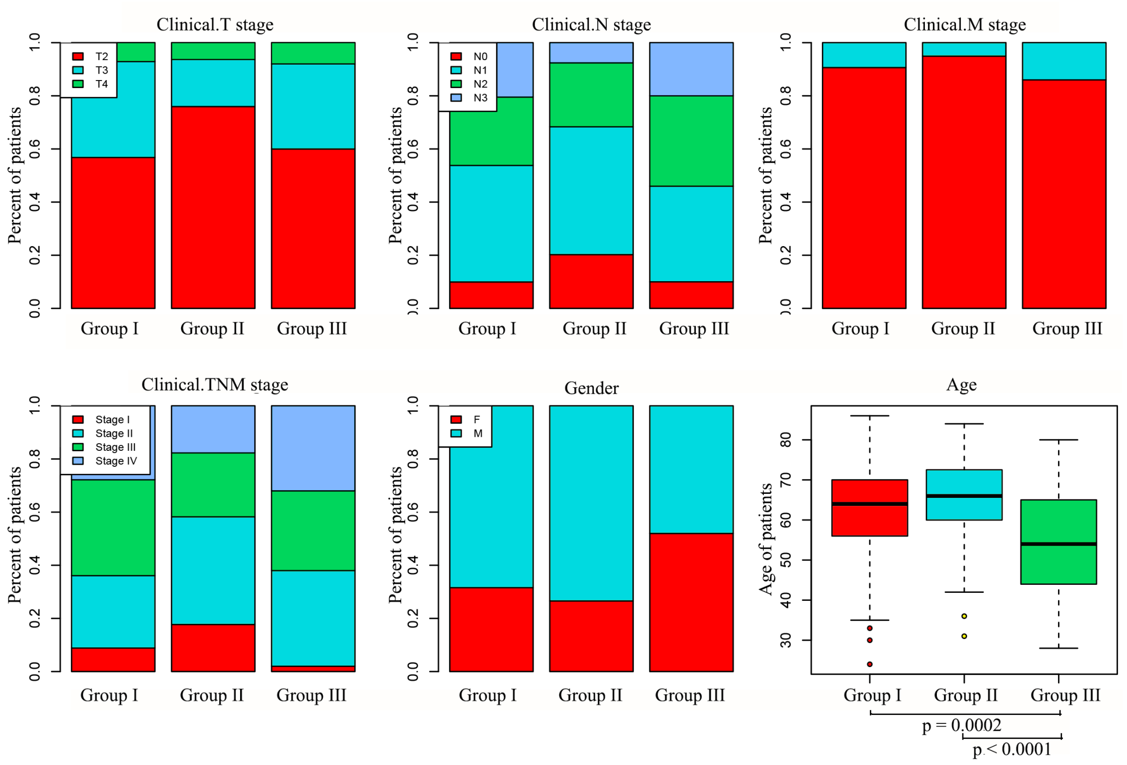

Supplementary Figure 5

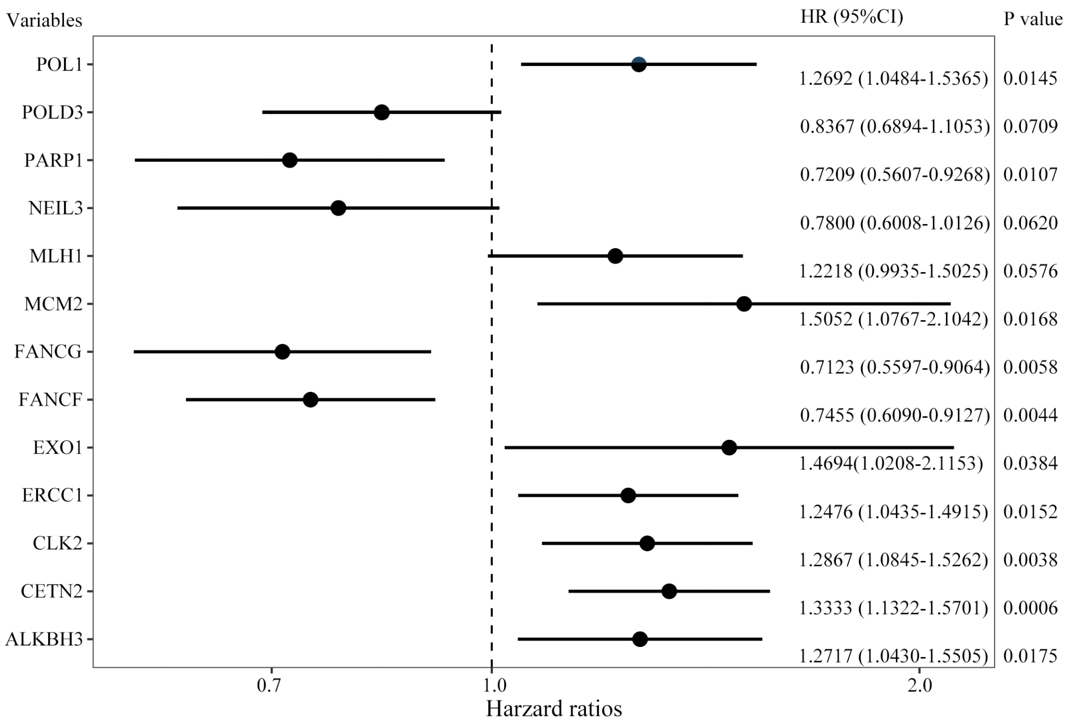

Supplementary Figure 6

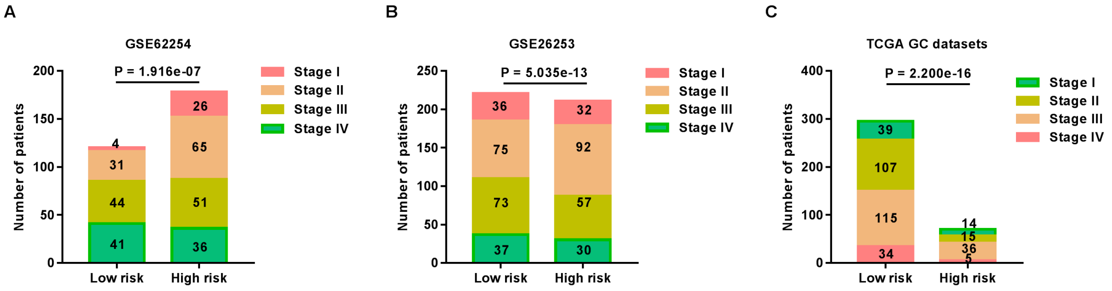

Supplementary Table 1 Information of all five GEO datasets

Study	GEO accession	Tumor samples	Normal samples	Platform
1	GSE33335	25	25	Affymetrix Human Exon 1.0 ST Array
2	GSE27342	80	80	Affymetrix Human Exon 1.0 ST Array
3	GSE63089	45	45	Affymetrix Human Exon 1.0 ST Array
4	GSE62254	300	0	Affymetrix Human Genome U133 Plus 2.0 Array
5	GSE26253	432	0	Illumina HumanRef-8 WG-DASL v3.0

Supplementary Table 2 List of 727 DNA repair genes

Gene size	Gene symbol
727	C17orf70, RPA32, hExoI, RPRGL4, UBE2V2, COCA2, COCA1, HYRC1, DNA-PKcs, XPAC, RNASEHI, PRIM1, PRIM2, XP3, XP1, MUS312, XPC, XPB, XPA, RNASEH1, XPG, XPF, XPE, XPD, STK1, HSPC150, XPV, SHFM1, SPGF4, PIG11, PIG16, HSU24186, TLAA, NEH2, NTHL1, FGP2, P1-CDC21, PMSL2, HMUDG, RAD4, RAD2, RAD1, MRP1, FA-D2, HR23A, HR23B, GLM3, SLX2A, WDR48, BACH1, CETN2, hFAN1, GTF2H, TTD, XFEPS, RAD30B, SPRTN, RAD30A, BRCC5, cdc19, BRCC2, BRCC1, BLM, CTC75, HMMH, CSB, HMG-1, hRAD54, CDKN7, TOP3, APITD1, TFB1, TFB2, TFB3, TFB4, TFB5, SOSS-B1, DNA2L, TTD1, TTD2, TTD3, POLQ, hNTH1, RNF53, POLZ, PRKDC, POLG, MUS81, POLE, POLD, POLB, p49, RP-A, POLN, CDC2, bHLHb39, POLK, POLI, POLH, mtSSB, PO-GA, REF1, MDG, bA120J8.2, RF-A, CHARAC17, hDNA2, DDB1, RIF1, DDBB, DDBA, ADPG, PRIM2A, TOP3B1, MEC1, GEN1, P85MCM, PPP1R128, GTF2H2C, KIAA1596, GTF2H2D, TTDA, SLX2B, RPA4, CHAF1A, CDC47, CDC46, RPA70, YHHQ1, PNCA3, PNCA2, HMGB1, PNCA4, CAK, MRXSC, UNG15, HEX1, IRIS, WRN, P58, FPG2, FPG1, p193, DCLRE1A, BLAP18, P52, GTF2H2C_2, PPP1R53, MDC1, VPARP, XPE-BF, CHRAC17, NBN, hFPG2, GIYD1, SEM1, DSS1, XPCE, ARTD4, ARTD2, ARTD3, XPCC, ARTD1, MO15, CALT, MBD4, LCFS2, UDG, DINB1, RP-A_p14, G22P1, FILS, KIAA1018, RAD10, RAD17, Mt-SSB, PADPRT-3, RAD18, FAAP90, NSX, SLX1A, HIGM4, SLX1B, hRad50, CCNL1, FAD2, FAD1, HRY, RNF66, KARP-1, p12, REV3L, p17, CENPS, NHEJ1, SBP-1, APX, ADPRT_1, COR1, OBFC2B, APE, RECQL5, RECQL4, P80, PPOL, RECQL3, RECQL2, XLF, MED1, DINP, SHPRH, TREX2, EXO1, P95, AYP1, ATRIP, DPE2, HCC5, MRE11, CSA, XTH2, CRA36.1, RFC, ECD, SSMED, BIVM-ERCC5, TELO2, H1RNA, SLX4, PEOB2, RAD502, RNASEH2A, RNASEH2C, RNASEH2B, hHR54, XPGC, NER-related, FAAP75, MCM4, NEIL1, NEIL3, NEIL2, MCM3, CLK2, ALKBH2, ALKBH3, FAAP95, BM28, C16orf75, RFC5, RFC4, ARMD5, ATM, RFC1, RFC3, RFC2, ATV, ATR, FANCD1, FANCD2, IRT1, BRIP1, MRE11B, SLX3, MRE11A, FANCS, FANCR, FANCQ, FANCP, DCLREC1C, FANCG, FANCF, FANCE, FANCD, FANCC, FANCB, FANCA, DGU, FANCO, FANCN, FANCM, FANCL, FANCJ, FANCI, FANCH, MTMR15, hTDG, PARP-2, PARP-1, PARP-4, p58, UNG, DUP, HAP1, DUT, SYCP3, pADPRT-1, P1CDC47, HsT16930, pADPRT-2, APNG, APEXL2, RAD52, RAD51, CDK7, XAB2, Shfdg1, ERCC8, POLA2, AAG, UVSSA, ERCC1, ERCC2, ERCC3, ERCC4, ERCC5, ERCC6, XAP1, p350, gs125, MDPL, RAD54A, RAD54B, hMLH1, RAD54L, DNPK1, FCTCS, POG, CDC54, hNEI3, BLAP75, FAAP250, BETAN, TTD-A, BROVCA4, BROVCA2, CUL-4B, P1.1-MCM3, BROVCA1, ENDOV, ZGRF2, ZGRF3, P62, p180, TOPBP1, P66, FACB, FACC, FACA, FACD, FACE, CUL4A, CMM6, CUL4B, UVSS1, UVSS2, D9, ADPRTL1, ADPRTL2, ADPRTL3, XP-V, MHCBFB, RAD9A, CDCL1, Mid1, FAAP43, ERCM2, Fanconi, KARP1, PARP, KMIN, HSAP, BRCAI, RS-SCID, PPP1R104, BRCA1, BRCA2, PNKP, PER1, GTBP,

KIAA1794, MRXS15, SMUG1, RBBP8, SFM2, COFS, IMD26, RBX1, RNHIA, EME1, EME2, PMS2CL, HELQ, SHFD1, FDG, RNF75, BS, XRCC9, XRCC1, XRCC3, XRCC2, XRCC5, XRCC4, XRCC7, XRCC6, HHR23B, HHR23A, MCG40308, PARPL, RFC140, RRM2B, CTCBF, CHEK2, CHEK1, DNAPK, PARP4, RAD50, NKGCD, PARP1, PARP2, PARP3, FAN1, MF1, HES-1, REV1, REV3, APTX, ERCC5-201, ERCC5-202, NTH1, TP53, BTF2, MITOTIN, UBE2A, PALB2, VWA5C, CAP35, POLE4, POLE3, POLE2, POLE1, HOGG1, HLTF, TOP3A, AT-V2, AT-V1, TOP3B, MAD2L2, p125, TEL2, ADPRT, FAP3, POLA1, AGS4, ATLD, SNM1C, MST075, BTBD12, HNPCC2, VAULT3, HNPCC1, HNPCC7, HNPCC4, HNPCC5, ATLD2, GTMBP, SCKL1, BTF2P44, hFPG1, USP1, Mis5, MSH2, P34, MSH3, MSH4, PMS2L3, MSH5, HSSB, YBL1, TGF2H5, MSH6, MRMV2, RAD51D, RAD51C, RAD51B, RAD51A, HNGS1, TRAD, PSCP, UBE2N, TDG, OCTS3, C9orf76, RP-A_p32, FAAP16, RP-A_p34, FAAP10, Ku86, TDT, UBE2B, RAD30, KUB2, RECA, ERCC11, DCLRE1B, DCLRE1C, RCC, DNA2, SHSF1, NUDT1, APEX1, FAH, APEX2, DMC1, FAB, FAC, FAA, FAF, FAG, FAD, FAE, PCNA, POL4P, PH5P, FRP1, FA2, FA3, CENPX, FA1, ROC1, FA4, MUTM, MCM7, MCM6, MCM5, RECC1, TP53BP1, MCM2, HES1, REV1L, EM9, RPA1, RPA3, RPA2, A-SCID, REC2, ADPRT3, ADPRT2, ADPRT1, UVDR, RFC37, SCIDA, HRAD51, HIGM5, R51H3, R51H2, BA554C12.1, HMG1, OGG1, HMG3, C19orf40, FAAP100, DNTT, APEX, AGS3, AGS2, CKN1, CKN2, TDP1, FA-H, MYH, DLEU8, LIG1, LIG2, LIG3, NEI1, NEI2, NEI3, APEN, HCAK, APE1, APE2, CEN2, RFC40, MGMT, PMS1, PMS2, ML8, TDP2, CENP-S, UBP, FA, OF, CENP-X, H2AFX, SPO11, P1-CDC46, RNF8, RNF4, CCNH, RLFB, RAD26, D3S3194, MAT1, FEN-1, FEN1, RNH1, LIG4, POLM, MMS19, POLL, RECQ2, CRCS10, CRCS12, UV20, GTF2H3, HYRC, UV-DDB2, UV-DDB1, NBS1, GTF2H1, p160, hCdc21, MRXHF2, UNG1, UNG2, UNG3, p39MO15, RECQL, GIYD2, TTDN1, p37, p34, MPG, anpg, RNHL, CDC21, TFIIH, RDH54, BROVCA3, RAD23B, RAD23A, RAD51L1, RAD51L2, RAD51L3, MUTYH, UBE2T, MHF1, CycH, MHF2, KU80, P1-MCM3, POLKAPPA, XRCC11, TREX1, NBSLD, NBS, HUS1, P1.h, POLD1, POLD2, POLD3, POLD4, KU70, COFS1, COFS2, COFS3, COFS4, SETMAR, APLF, CAK1, DDB2, POLDS, OGH1, HNPCC, MNAT1, ZGRF7, POLA, HsRad51, PIG50, SCKL, HR54, P68, FANCT, UAF1, P105MCM, JUNB, SCP3, MMS4L, STRA13, RDM1, p44, RMI1, RMI2, PHF9, SSBP, RFC38, FCC1, FCC2, PRPF19, MLH4, MLH3, RFC36, MLH1, A1, Pol_Mu, FAAP20, FAAP24, Tdt-N, RAD25, T-BTF2P44, GTF2H2, LIG4S, GTF2H5, GTF2H4, REPA2, SSBP1, SPG60, NKCD, PNAS146, C6orf175, RNF168, NFIV, REPA3, RAD3OB, REPA1, HHL

Supplementary Table 3 Enrichment results of DNA repair genes in three GEO datasets by GSEA GO_BP

DATASET	GO_BP	ES	P value
GSE63089	regulation of protein modification process	-0.6373	0.0040
	cell cycle phase transition	-0.583	0.0408

	DNA metabolic process	-0.7703	0.0337
	cell cycle G1/S phase transition	-0.5563	0.0488
	mitotic recombination	-0.5564	0.0323
	positive regulation of molecular function	-0.5139	0.0317
	response to oxygen containing compound	-0.5639	0.0425
	regulation of cell proliferation	-0.5953	0.0257
	DNA synthesis involved in DNA repair	-0.5306	0.0396
	regulation of intracellular signal transduction	-0.5074	0.0481
	recombinational repair	-0.539	0.0383
	DNA biosynthetic process	-0.4911	0.0363
GSE33335	positive regulation of molecular function	-0.6137	0.0395
	positive regulation of catalytic activity	-0.6315	0.0487
GSE27342	cell cycle	-0.6086	0.0000
	cell cycle process	-0.5929	0.0040
	DNA strand elongation involved in DNA replication	-0.6204	0.0021
	DNA dependent DNA replication	-0.5638	0.0244
	DNA strand elongation	-0.5764	0.0061
	DNA metabolic process	-0.7398	0.0383
	DNA replication	-0.5234	0.0202
	mitotic cell cycle	-0.5251	0.0120
	cell cycle checkpoint	-0.5294	0.0180
	meiotic cell cycle	-0.4875	0.0392
	cell cycle G1/S phase transition	-0.5642	0.0266
	cell cycle phase transition	-0.556	0.0310
	DNA integrity checkpoint	-0.5016	0.0264
